# A2AR-mediated CXCL5 upregulation on macrophages promotes NSCLC progression via NETosis

**DOI:** 10.1007/s00262-024-03689-3

**Published:** 2024-04-20

**Authors:** Qingyang Lei, Shanshan Zhen, Lei Zhang, Qitai Zhao, Li Yang, Yi Zhang

**Affiliations:** 1 Biotherapy Center and Cancer Center, The First Affiliated Hospital of Zhengzhou University, 1 Jianshe East Road, Zhengzhou, 450052 Henan China; 2 State Key Laboratory of Esophageal Cancer Prevention & Treatment, Zhengzhou University, Zhengzhou, China; 3 Henan Key Laboratory for Tumor Immunology and Biotherapy, Zhengzhou, China; 4 Thoracic Surgery Department, The First Affiliated Hospital of Zhengzhou University, Zhengzhou, China

**Keywords:** TAMs, A2AR, Lung cancer, Immunotherapy

## Abstract

Tumor-associated macrophages (TAMs) are abundant in tumors and interact with tumor cells, leading to the formation of an immunosuppressive microenvironment and tumor progression. Although many studies have explored the mechanisms underlying TAM polarization and its immunosuppressive functions, understanding of its progression remains limited. TAMs promote tumor progression by secreting cytokines, which subsequently recruit immunosuppressive cells to suppress the antitumor immunity. In this study, we established an in vitro model of macrophage and non-small cell lung cancer (NSCLC) cell co-culture to explore the mechanisms of cell-cell crosstalk. We observed that in NSCLC, the C-X-C motif chemokine ligand 5 (CXCL5) was upregulated in macrophages because of the stimulation of A2AR by adenosine. Adenosine was catalyzed by CD39 and CD73 in macrophages and tumor cells, respectively. Nuclear factor kappa B (NFκB) mediated the A2AR stimulation of CXCL5 upregulation in macrophages. Additionally, CXCL5 stimulated NETosis in neutrophils. Neutrophil extracellular traps (NETs)-treated CD8^+^ T cells exhibited upregulation of exhaustion-related and cytosolic DNA sensing pathways and downregulation of effector-related genes. However, A2AR inhibition significantly downregulated CXCL5 expression and reduced neutrophil infiltration, consequently alleviating CD8^+^ T cell dysfunction. Our findings suggest a complex interaction between tumor and immune cells and its potential as therapeutic target.

**Supplementary Information:**

The online version contains supplementary material available at 10.1007/s00262-024-03689-3.

## Introduction

Non-small cell lung cancer (NSCLC) is the most prevalent type of lung cancer. Immunotherapies, especially PD1 and PDL1 inhibitors, have been approved as first-line therapy for NSCLC. Despite advancements, the overall response rate is limited because of resistance to therapy. Previous studies have delved into antitumor immunity mechanisms to boost therapeutic effects, highlighting the crucial role of the immunosuppressive environment in therapy resistance. Studies reveal that in PD1/PDL1 therapy-resistant patients, tumor-associated macrophages (TAMs) impede antitumor immunity and foster resistance to PD1/PDL1 therapy [1, 2].

Macrophages in the tumor microenvironment (TME) constitute approximately 50% of the infiltrating immune cells. Despite attempts to enhance antitumor immunity by targeting TAMs, their plasticity in tumors complicates these efforts [3]. TAMs contribute to T cell dysfunction and exclusion through cell-to-cell interactions driven by various soluble factors, including metabolites and cytokines [4]. Extracellular adenosine, a CD39 and CD73-catalyzed ATP metabolite, suppresses antitumor immunity by binding to the adenosine receptors on immune cells [5]. A2AR, a high-affinity adenosine receptor expressed by TAMs, favors type 2 macrophage polarization, contributing to tumor progression [6, 7].

Neutrophils respond to cytokines to enter the tumor microenvironment and release neutrophil extracellular traps (NETs) that can promote cancer cell metastasis [8–10]. Yet, its impact on anti-tumor immunity remains unclear.

TAMs are important sources of cytokines in the TME [11–14]. In our study, we found that CD73 in NSCLC tumor cells and CD39 in macrophages led to extracellular adenosine accumulation in TME, A2AR activation and CXCL5 secretion in macrophages. CXCL5 recruited neutrophils and triggered NETs that inhibited CD8^+^ T cell function. A2AR inhibition in mouse tumors reduced CXCL5 expression, decreased NETs, and enhanced CD8^+^ T cell function. These findings suggest that blocking A2AR signaling could regulate TAMs and tumor cell crosstalk, presenting a potential strategy for improving antitumor immunity.

## Methods

### Healthy donors and patient samples

Peripheral blood samples were obtained from healthy donors recruited from the Henan Red Cross Blood Center with informed consent. Lung tumor samples for immunofluorescent staining were collected from untreated patients with NSCLC and surgically resected the specimen at the First Affiliated Hospital of Zhengzhou University with Ethics Committee approval. Patients provided informed consent or relative's assent.

### Cell lines

H460 and A549 NSCLC cell lines, obtained from the Chinese Academy of Sciences Shanghai Branch Cell Bank, were cultured in RPMI 1640 and DMEM-F12 with 10% FBS at 37 °C, 5% CO_2_.

### Mouse model

C57BL/6J mice (6–8 weeks) obtained from Beijing Vital River Biocytogen were housed under specific pathogen-free conditions. Humane care followed the Guide for the Care and Use of Laboratory Animals (National Institutes of Health publication 86–23, revised 1985). Lewis lung cancer (LLC) cells (1 × 10^6^) were subcutaneously injected for treatment evaluation, and tumor growth was monitored weekly using PerkinElmer IVIS spectrum until day 28. Tumor-infiltrating immune cells were analyzed after 2 × 10^6^ LLC cells were injected, and the mice were sacrificed on day 21.

### Co-culture of tumor cells and macrophages

Healthy donor-derived macrophages (5 × 10^5^/well) and NSCLC cells (H460/A549, 8 × 10^5^/well) were co-cultured in a Transwell device (0.4um). CPI-444 (Selleck, S6646), sodium metatungstate (POM-1) (Selleck, S5525), and JSH-23 (Selleck, S7351) were added 2 h before 24 h incubation. The controls included individual cultures at matching densities. Supernatants and cells were collected for protein and mRNA analysis.

### RNA-seq analysis

RNA was extracted from human macrophages and T cells using RNAiso Plus (Takara, 9109). The mRNA sequencing and differential expression analyses were conducted at the Beijing Genomics Institute. The differential expression genes are represented in Supplementary Table 1.

### Enzyme-linked immunosorbent assay (ELISA)

CXCL5 concentration in the cell culture supernatant was quantified using an ELISA kit (BioLegend, CAT#440,904) according to the manufacturer’s protocol.

### Western blot

Cells were lysed in RIPA buffer and sonicated. Proteins were separated using 12% SDS-PAGE gel and transferred to nitrocellulose membranes. The membranes were blocked with 5% defatted milk for 1 h, incubated with primary antibodies (1:1000) overnight, and then with HRP-conjugated secondary antibodies for 1 h. Protein signals were visualized using ECL detection reagents.

### Immunofluorescent staining

Tumor tissues, fixed in 4% paraformaldehyde, were paraffin-embedded to generate 5 μm sections. Antigen retrieval was performed using citrate solution. Sections were permeabilized (0.1% Triton X-100), blocked (5% BSA), and incubated overnight at 4 °C with primary antibody. Sections were incubated with fluorescence-conjugated secondary antibodies (1 h at room temperature), mounted with DAPI-containing medium, imaged using Olympus microscope and Vectra Automated Multispectral Imaging system, and analyzed with ImageJ software. The dilution rates are represented in Table 1.

**Table 1:** Primary and secondary antibody dilution rates

Primary antibody	Dilution rate	Cat
Anti-human CXCL5	1:100	R&D, AF254
Anti-mouse LIX(CXCL5)	1:200	R&D, MAB433
Anti-mouse CD8	1:200	Absin, abs120101
Anti-mouse F4/80	1:200	CST, 70,076
Anti-mouse citH3	1:200	Absin, abs153262
Anti-mouse Ly6G	1:200	CST, 31469 s
Anti-A2AR	1:200	Novusbio, NBP1-39,474

Secondary antibody	Dilution rate	Cat
Anti-goat AF594	1:5000	Jackson, 711–545-150
Anti-rabbit AF488	1:5000	Jackson, 711–545-152
Anti-rabbit AF647	1:5000	Jackson, 711–585-152
Anti-goat AF647	1:5000	Jackson, 805–605-180

**Table 2:** Antibodies used for human antigens

Antibody	Cat
CD14	BioLegend, 325620
CD163	BioLegend, 333605
A2AR	Novusbio, NBP1-39474
CD8	BioLegend, 344714
TIM3	BioLegend, 364805
LAG3	BioLegend, 369309
IFN-γ	BioLegend, 502512
TNF-α	BioLegend, 502936
IL2	BioLegend, 500348
Phospho-p65	CST, 3031
Anti-rabbit AF488	Jackson, 711–545-152

**Table 3:** Antibodies used for mouse antigens

Antibody	Cat
CD8	BioLegend, 100714
Ki67	BioLegend, 151215
IL2	BioLegend, 503808
PD1	BioLegend, 135225
TIM3	BioLegend, 119723
IFN-γ	BioLegend, 505832

### Tissue microarrays (TMA)

TMA (HLug-NSCLC150PT-01) from Shanghai Outdo Biotech contained 75 NSCLC and paired para-tumor tissues. Immunohistochemistry was performed by Wuhan Servicebio company. Clinical–pathological parameters are shown in supplementary Table 2.

### Lentivirus transfection

Stable ShCD73-expressing H460 and A549 cell lines were generated by using lentiviral transduction and antibiotic selection. The shCD73 plasmid (hU6-MCS-Ubiquitin-firefly_Luciferase-IRES-puromycin) was purchased from GeneChem. JetPRIME^®^ Kit was used to transfect plasmids into HEK 293 T cells. After 48 h, the supernatant containing lentivirus was added to the A549 and H460 plates with 6 ng/ml Polybrene (Solarbio, H8761). Puromycin (MCE, HY-B1743A, 2 μg/ml) was added after 48 h for shCD73-expressing cell selection.

### Flow cytometry and imaging flow cytometry

Cells were stained with fluorescent-conjugated antibodies (15 min) or primary antibodies (30 min) at 4 °C in 1% FBS flow buffer. For intracellular staining, cells were fixed in 4% paraformaldehyde (Servicebio, CAT# G1101), permeabilized (BioLegend, CAT# 421,002; BD, CAT#562,574), and stained with antibodies (15 min) at 4 °C. Data were acquired with a Beckman Coulter DxFLEX cytometer. Analysis was performed using the CytExpert IDEAS Application, FlowJo v10, and CytExpert. The antibodies used are listed in Tables 2 and 3. Standard and imaging flow cytometry data were acquired and analyzed accordingly.

### Quantitative real-time PCR (qRT-PCR)

Total RNAs were extracted using TRIZOL (Takara, CAT#9101) following the manufacturer’s instructions. The RNA concentrations were measured using a NanoDrop 2000 (Thermo Fisher Scientific, USA). Next, 1 µg of RNA was reverse transcribed to cDNA using HiScript III RT SuperMix for qPCR (+ gDNA wiper) (Vazyme, CAT# R223-01). Real-time PCR (40 cycles, annealing temperature 60 °C) was performed using the ChamQ Universal SYBR qPCR Green Master Mix (Vazyme, CAT# Q711-02) on a CFX96 real-time system (Bio-Rad, USA). Relative gene expression was quantified by the 2 − ΔΔCT method, and human β-actin served as an internal control for each reaction. The primers used for qPCR are listed in Supplementary Table 3.

### Neutrophil isolation and NETs stimulation

Neutrophils were isolated from fresh peripheral blood, according to the manufacturer’s instructions (P9040; Solarbio). Neutrophils were collected and washed. The residual red blood cells were lysed (R1010; Solarbio). Neutrophils were seeded into 10 cm culture plates and stimulated with PMA (100 ng/ml) for 4 h. NETs in the supernatant were collected and centrifugated at 10,000*g* for 15 min at 4 °C, seeded in 48-well plates, and incubated overnight.

CD8^+^ T cells were activated 3 days before coculturing with NETs using CD3 (5 μg/ml) and CD28 (2.5 μg/ml) antibodies and rhIL2 (100 IU). CD8^+^ T cells (1 × 10^6^ per well) were treated with or without NETs for 3 days and collected for flow cytometry and RNA-seq.

### Dual-luciferase reporter assay

293T cells were transfected with 0.25 μg luciferase plasmid pGL3-CXCL5, 0.25 μg pcDNA3.1-RELA reporter plasmid and 500 ng Renilla plasmid pRL-TK for 48 h. Cell lysates were collected and analyzed using the Dual-Luciferase Reporter Gene Assay Kit (YEASEN, Cat: 11402ES60) according to the manufacturer’s instructions. Luciferase and Renilla bioluminescence were detected using SpectraMax^®^ iD3 Multi-Mode Microplate Reader. Firefly luciferase activity was normalized to the Renilla luciferase activity.

### Bioinformatics analysis

Public gene expression data for The Cancer Genome Atlas (TCGA) lung adenocarcinoma (LUAD) and lung squamous cell carcinoma (LUSC) were acquired to analyze gene correlation using the online websites GEPIA (http://gepia.cancer-pku.cn) and TIMER [15] (https://cistrome.shinyapps.io/timer/) and analyzed using Pearson’s correlation test. TISIDB was used to analyze the correlation between cell abundance and CXCL5 expression, and the correlation between immune subtype and CXCL5 expression [16]. The gene matrix used to identify neutrophils and macrophages was selected from the study by Charoentong et al. [17]. The adenosine signature dataset, previously reported in a renal cell carcinoma study [18], was used to analyze the RNA-seq data. The signature score was calculated as the mean log2 (TPM + 1) value of each gene in the differentially expressed gene (DEG) dataset. Heatmap and KEGG enrichment analyses were performed using the OmicShare Tool (GENE DENOVO, https://www.omicshare.com). GSEA was performed using GSEA 4.3.2 and compared with the exhaustion signature (Supplementary Table 4) described in two previous studies. For survival analysis, dataset (GSE8894) was collected and analyzed using PrognoScan (http://dna00.bio.kyutech.ac.jp/PrognoScan/).

### Statistical analysis

Statistical analyses were performed using GraphPad Prism 9 software (GraphPad Software, USA). In the bar graphs, data were shown as mean ± SD and analyzed by two-tailed student’s *t* test or one-way ANOVA with Tukey’s test. Statistical significance was set at *P* < 0.05. *P* values are represented as follows: **** *P* < 0.0001, *** *P* < 0.001, ** *P* < 0.01, and * *P* < 0.05.

## Results

### NSCLC cells stimulate CXCL5 upregulation on macrophages

**Fig. 1:**
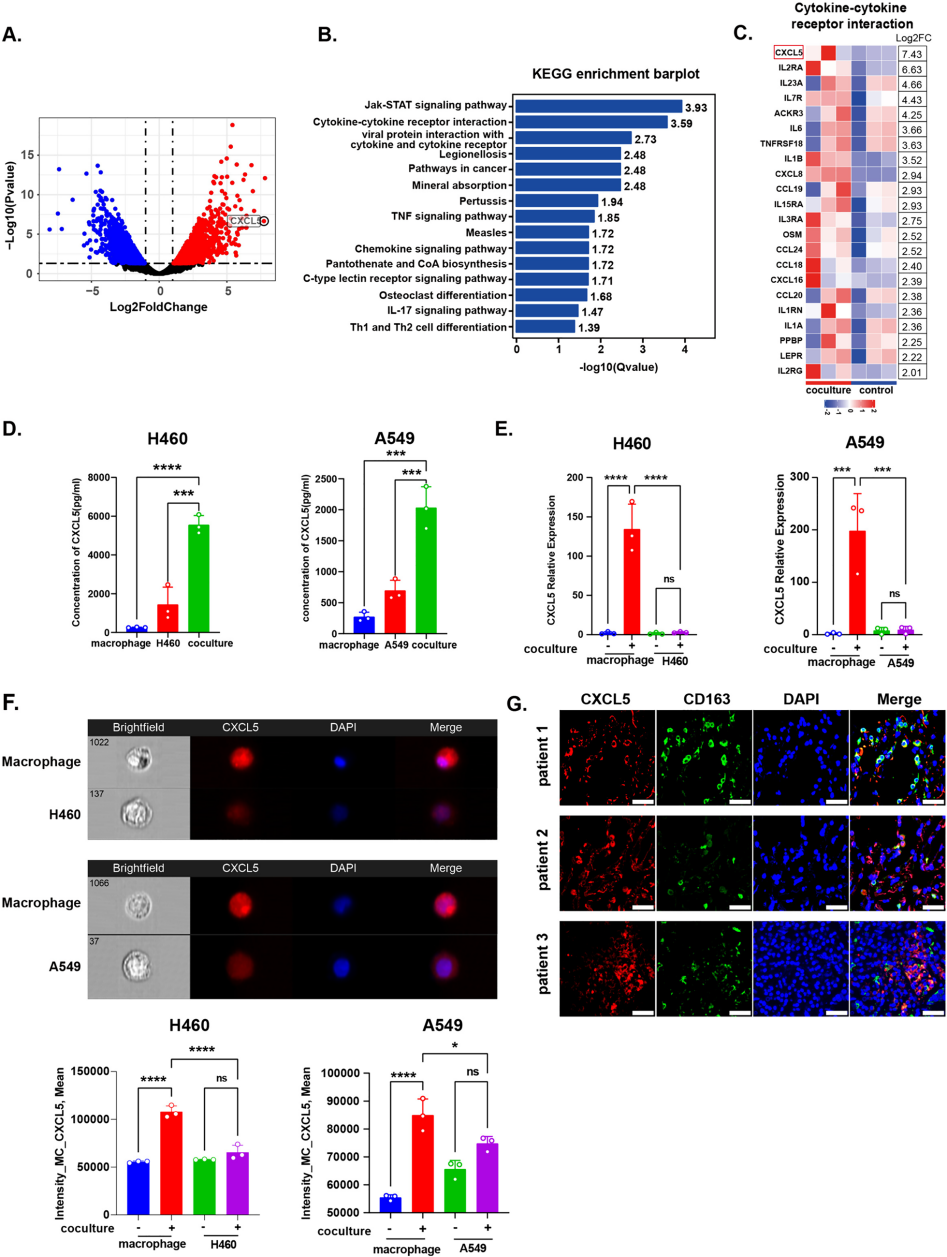
CXCL5 expression in macrophages and NSCLC cells. **a** Volcano plot of DEGs from untreated vs co-cultured macrophages. **b** Bar plot of KEGG enrichment pathway analyzing DEGs. **c** Heatmap of genes enriched in cytokine-to-cytokine receptor interaction. **d** CXCL5 concentration in supernatant generated from macrophages, NSCLC cell lines and the co-culture system detected by ELISA. **e** RT-PCR detecting CXCL5 relative expression in untreated or co-cultured macrophages, H460 and A549. **f** CXCL5 expression in co-cultured macrophages and NSCLC cell lines. **g** CXCL5 and macrophage localization in tumor tissues of patients with NSCLC (scale bar: 100 μm). All data are mean ± SD. **d**, **e**, and **f** were analyzed by one-way ANOVA with Tukey’s test. Data are cumulative results from at least three independent experiments

The interaction between the immune system and tumor cells is key for tumors to manipulate the TME and escape immune elimination. To explore the mechanisms of TAMs and tumor cell interactions in NSCLC, we mimicked the tumor microenvironment by co-culturing NSCLC cell line H460 with macrophages induced in vitro using Transwell devices for 24 h (Supplementary Fig. 1A, B). We conducted RNA-seq on macrophages and H460 cells before and after co-culture (Supplementary Fig. 1C, 2). We analyzed differentially expressed genes in macrophages (Supplementary Table 1) and observed that DEGs were enriched in cytokine-receptor-related KEGG pathways (Fig. 1a, b). The cytokine CXCL5 was the most varied gene, with a Log2FC of approximately 7 compared with the control after co-culture (Fig. 1c). We compared the CXCL5 concentrations in the co-culture, macrophage, and H460 cell supernatants and found the highest CXCL5 concentration in the co-culture supernatant (Fig. 1d). Analysis of TCGA data showed that macrophage abundance was associated with CXCL5 expression in tumor tissues (Supplementary Fig. 1E), suggesting an association between macrophages and CXCL5. RT-PCR confirmed that the relative expression of CXCL5 in macrophages after co-culture was much higher than that in H460 and A549 cells before and after co-culture (Fig. 1e), indicating that the upregulation of CXCL5 mainly originated from macrophages. Imaging flow cytometry of H460, A549 and macrophages after co-culture demonstrated that macrophages generated more CXCL5 than H460 and A549 cells (Fig. 1f). Furthermore, the detection of CXCL5 in tumor tissues from patients with NSCLC demonstrated that TAMs expressed higher levels of CXCL5 than other cells (Fig. 1g). These results demonstrated that CXCL5 expression is upregulated in macrophages, suggesting that the interaction between tumor cells and macrophages is mediated by tumor cell-derived factors.

### The adenosine signature is associated with CXCL5 upregulation

**Fig. 2:**
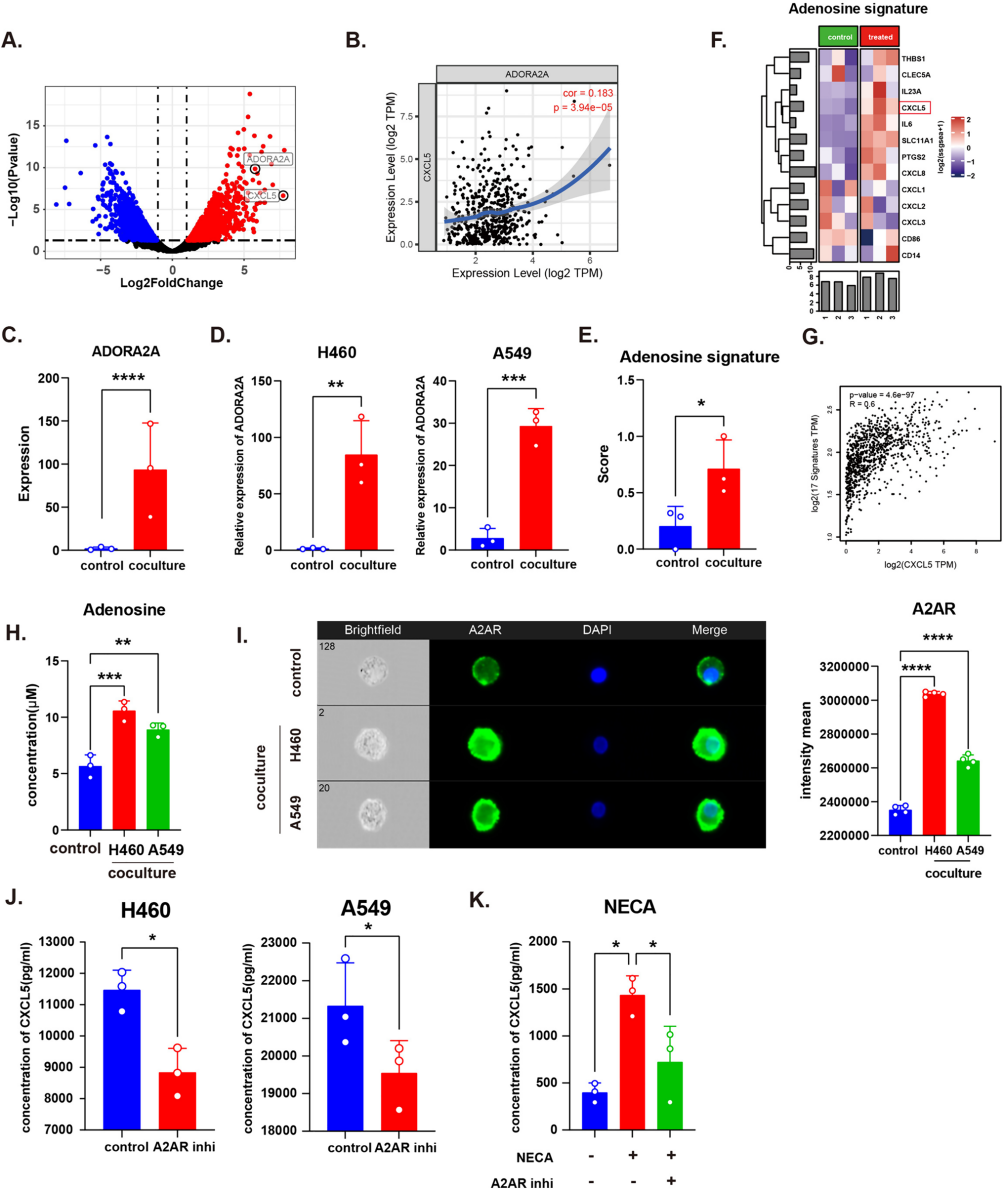
Adenosine receptor A2AR regulate CXCL5 expression on macrophages.** a** Volcano plot of ADORA2A and CXCL5. **b** Analysis of ADORA2A and CXCL5 correlation in patients with LUSC in the TCGA database. **c**, **d**. ADORA2A expression in untreated and co-cultured macrophages was detected using RNA-seq and RT-PCR. **e**, **f**. Adenosine signature in untreated and co-cultured macrophage RNA-Seq. **g** Correlation of adenosine signature and CXCL5 in TCGA NSCLC data. **h** Adenosine concentration of macrophage and co-culture supernatants. **i** A2AR expression on untreated and co-cultured macrophages detected by imaging flow cytometry. **j** CXCL5 concentration in control and A2AR inhibitor (CPI-444, 10μM)-treated supernatants. **k** CXCL5 concentration in macrophage supernatant after stimulation with NECA (1μM) and/or A2AR inhibitor (CPI-444, 10 μM). All data are mean ± SD. **c**, **d**, and **e** were analyzed by one-way ANOVA with Tukey’s test. **h**–**k** were analyzed by two-tailed, unpaired Student’s *t* test. Data are cumulative results from at least three independent experiments

As the results showed that macrophages were educated by tumor cells and consequently generated more CXCL5, we further analyzed RNA-seq data. By exploring the correlation between CXCL5 and the top 10 DEGs (Supplementary Table 5), we observed that CXCL5 was positively correlated with ADORA2A, G0S2, MARCO, MT1E, and SNAI1 in the TCGA database (Fig. 2a, b, Supplementary Fig. 3A, B). Since the co-culture device we used was built for indirect co-culture, we assumed that soluble factors might mediate the interaction between tumor cells and macrophages and that proteins localized on the cell membrane were more likely to be directly responsible for the upregulation of CXCL5 in macrophages. Therefore, MT1E, G0S2, and SNAI1 were excluded from consideration because studies have shown that their coding proteins are localized in the plasma and nucleus [19–21]. Both MARCO and ADORA2A encode for membrane proteins. However, A2AR (encoded by ADORA2A) which is the receptor for extracellular adenosine has been reported to be associated with cytokine secretion in monocytes [18]. Further experiments confirmed that ADORA2A was upregulated after co-culture (Fig. 2c, d, Supplementary Fig. 4B, C). We then analyzed the adenosine signature that was upregulated after stimulating A2AR [18]. The adenosine signature score, which represents the activation of A2AR signaling, was higher in macrophages after co-culture, as was the cAMP metabolic process enrichment score (Fig. 2e, f, Supplementary Fig. 4A). Analysis of TCGA database revealed that the adenosine signature was positively associated with CXCL5 expression in NSCLC (Fig. 2g). We detected an increase in adenosine concentration in the co-culture supernatants (Fig. 2h). A2AR expression was upregulated according to the imaging flow cytometry results (Fig. 2i). By applying CPI-444, a selective A2AR inhibitor, to the co-culture medium, we observed the downregulation of CXCL5 (Fig. 2j). Treating macrophages with adenosine analog 5ʹ-N-Ethylcarboxamidoadenosine (NECA) significantly stimulated CXCL5 generation, while CPI-444 was able to reduce the CXCL5 expression  (Fig. 2k). These results suggest that tumor cell-induced A2AR signaling is responsible for CXCL5 upregulation in macrophages.

### The discriminative expression of CD39 and CD73 on macrophages and tumor cells together regulates CXCL5 expression

**Fig. 3:**
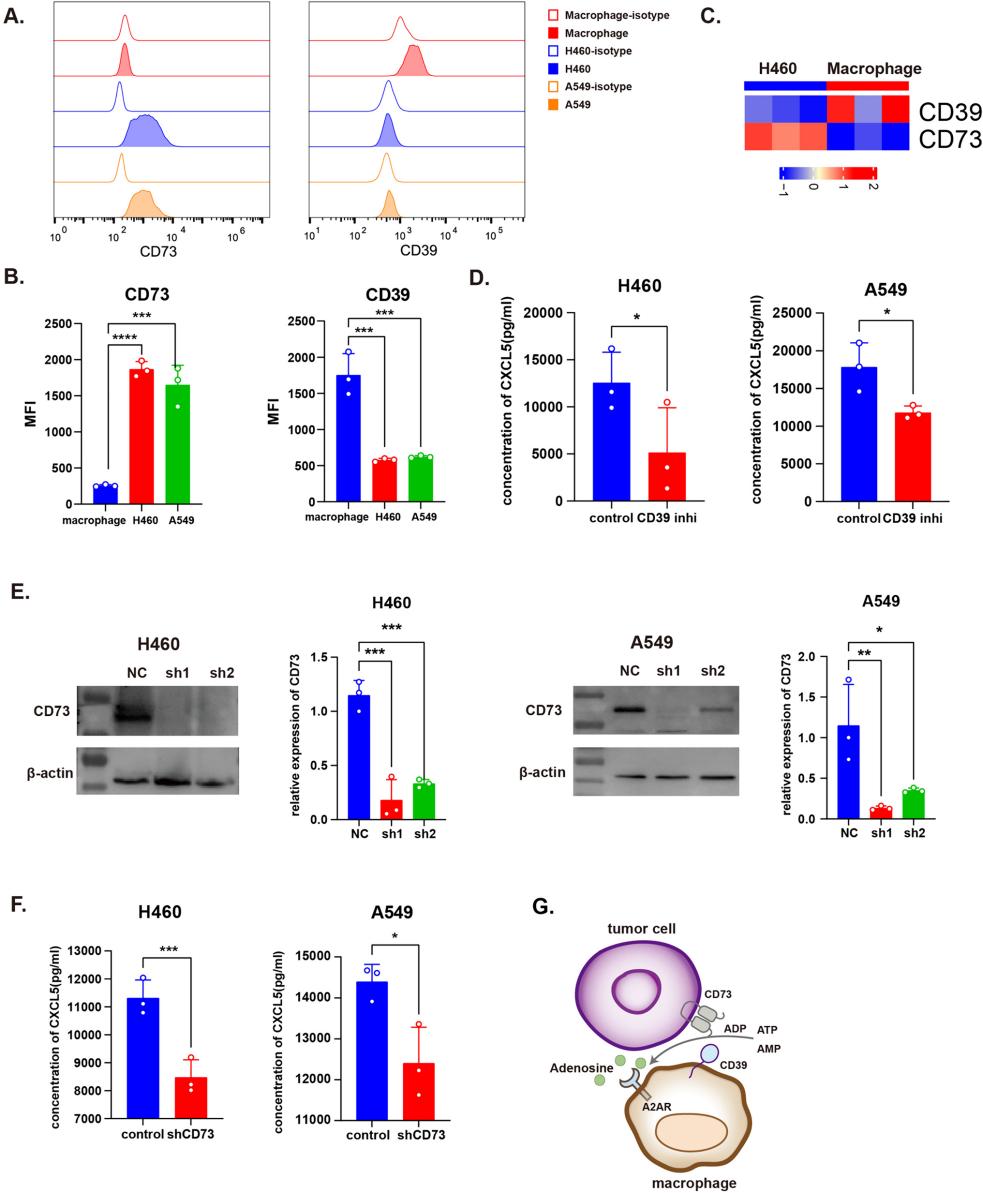
Expression of CD39 and CD73 on macrophages and NSCLC cells regulated CXCL5 expression on macrophages. **a** CD39 and CD73 expression in macrophages, H460 and A549 was detected by flow cytometry. **b** MFI of CD39 and CD73 on macrophages H460 and A549. **c** CD73 expression of H460 shNC/CD73 and A549 shNC/CD73 cells. **d** CXCL5 concentration of co-culture supernatant without or with the presence of CD39 inhibitor (POM-1, 10μM). **e** CD73 expression detected by western blot and RT-PCR. **f** CXCL5 concentration of co-culture supernatant without or with CD73 knockdown in H460 and A549. **g** Diagram showing the discriminative expression of CD39 and CD73 on macrophages and tumor cells. All data are mean ± SD. **b** and **e** were analyzed by one-way ANOVA, while **d** and **f** were analyzed by two-tailed, unpaired Student’s *t* test. Data are cumulative results from at least three independent experiments

Extracellular adenosine is a ligand of A2AR, which can be generated from ATP hydrolysis driven by CD39-CD73 catalysis. We detected higher CD39 expression in macrophages and higher CD73 expression in tumor cells by comparing the two groups using flow cytometry and RNA-seq (Fig. 3a–c); flow cytometry demonstrated that CD39 and CD73 were only expressed in macrophages and tumor cells, respectively (Fig. 3a, b). These results suggested that cooperation between macrophages and tumor cells is necessary to promote extracellular adenosine generation. As expected, CXCL5 concentration decreased after CD39 inhibition (Fig. 3d). We then constructed CD73-knockdown NSCLC cell lines H460-shCD73 and A549-shCD73 and selected the sh1 RNA-knockdown cell line for further experiments (Fig. 3e). The CXCL5 concentration decreased when macrophages were co-cultured with shCD73 cells instead of shNC cells (Fig. 3f). These results indicated that cooperation between macrophages and tumor cells is required for extracellular adenosine accumulation and CXCL5 upregulation (Fig. 3g).

### Transcription factor NFκB mediates the A2AR-induced CXCL5 upregulation

**Fig. 4:**
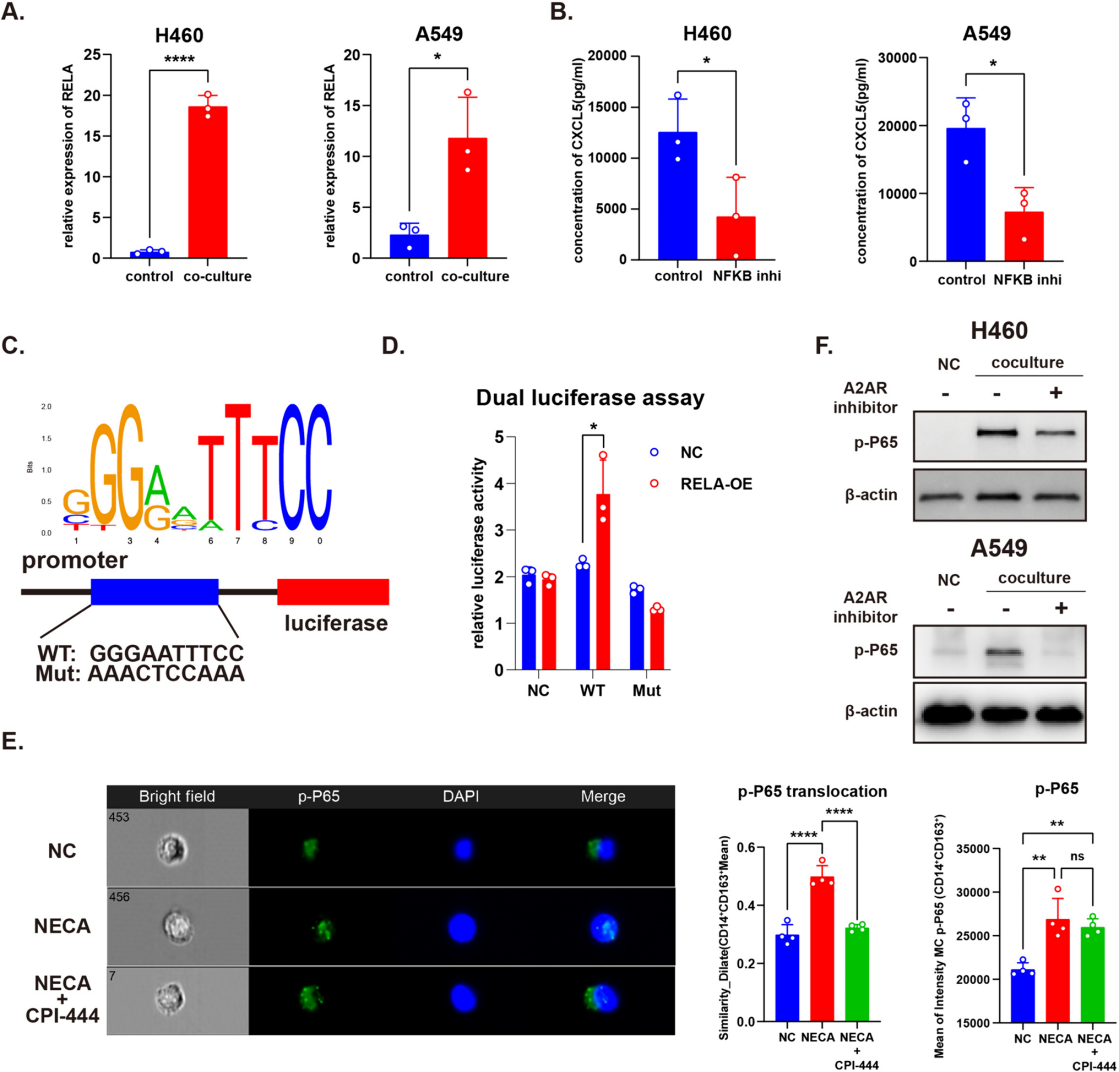
NFκB regulates macrophage CXCL5 expression. **a** Relative expression of RELA in untreated and co-cultured macrophages. **b** CXCL5 concentration of co-culture supernatant treated with or without the presence of NFκB inhibitor (JSH-23, 10 μM). **c** Putative binding site for RELA in the CXCL5 promoter region. **d** Dual-luciferase reporter assay detecting binding of RELA to the CXCL5 promoter region. **e** Translocation of phosphorylated P65 after A2AR stimulation by NECA (1 μM). **f** Phosphorylated P65 (RELA) expression in macrophages and co-cultured macrophages, with or without A2AR inhibitor treatment. All data are mean ± SD and were analyzed by two-tailed, unpaired Student’s t test, except data in **e** were analyzed by one-way ANOVA with Tukey’s test. Data represent the cumulative results from at least three independent experiments

We identified transcription factors involved in A2AR-induced CXCL5 upregulation. According to the RNA-seq data, certain transcription factors were upregulated in macrophages (Supplementary Table 6). We cross-checked this gene list against the transcription factor list of genes predicted by the online tool PROMO (https://alggen.lsi.upc.es/cgi-bin/promo_v3/promo/promoinit.cgi?dirDB=TF_8.3) (Supplementary Table 6), which had binding sites located in the CXCL5 promoter sequence and found seven transcription factors in both lists (STAT4, ETS2, REL, RELA, JUNB, ETS1, and CEBPB). We examined these transcription factors using JASPAR (https://jaspar.genereg.net), and RELA (encoding P65, a subunit of NFκB) had the highest relative score (Supplementary Table 6). Other studies have reported that RELA is a transcription factor for CXCL5 [22]. Therefore, we used RT-PCR and western blotting to verify the upregulation of RELA and phosphorylated P65 in macrophages after co-culture (Fig. 4a, f). By adding the NFκB inhibitor JSH-23 to the co-culture medium, we confirmed that NFκB regulated CXCL5 concentration (Fig. 4b). The dual-luciferase reporter assay showed that RELA was bind to the promoter region of CXCL5 (Fig. 4c, d). We also observed that phosphorylated P65 (RELA) was upregulated in post-co-cultured macrophages and translocated from the cytoplasm to the nucleus of macrophages after A2AR agonist stimulation. However, the A2AR inhibitor inhibited this upregulation and translocation (Fig. 4e, f). These results indicated that transcription factor NFκB mediated A2AR-regulated CXCL5 upregulation.

### CXCL5 stimulates neutrophil NETosis which promotes CD8^+^ T cell dysfunction

**Fig. 5:**
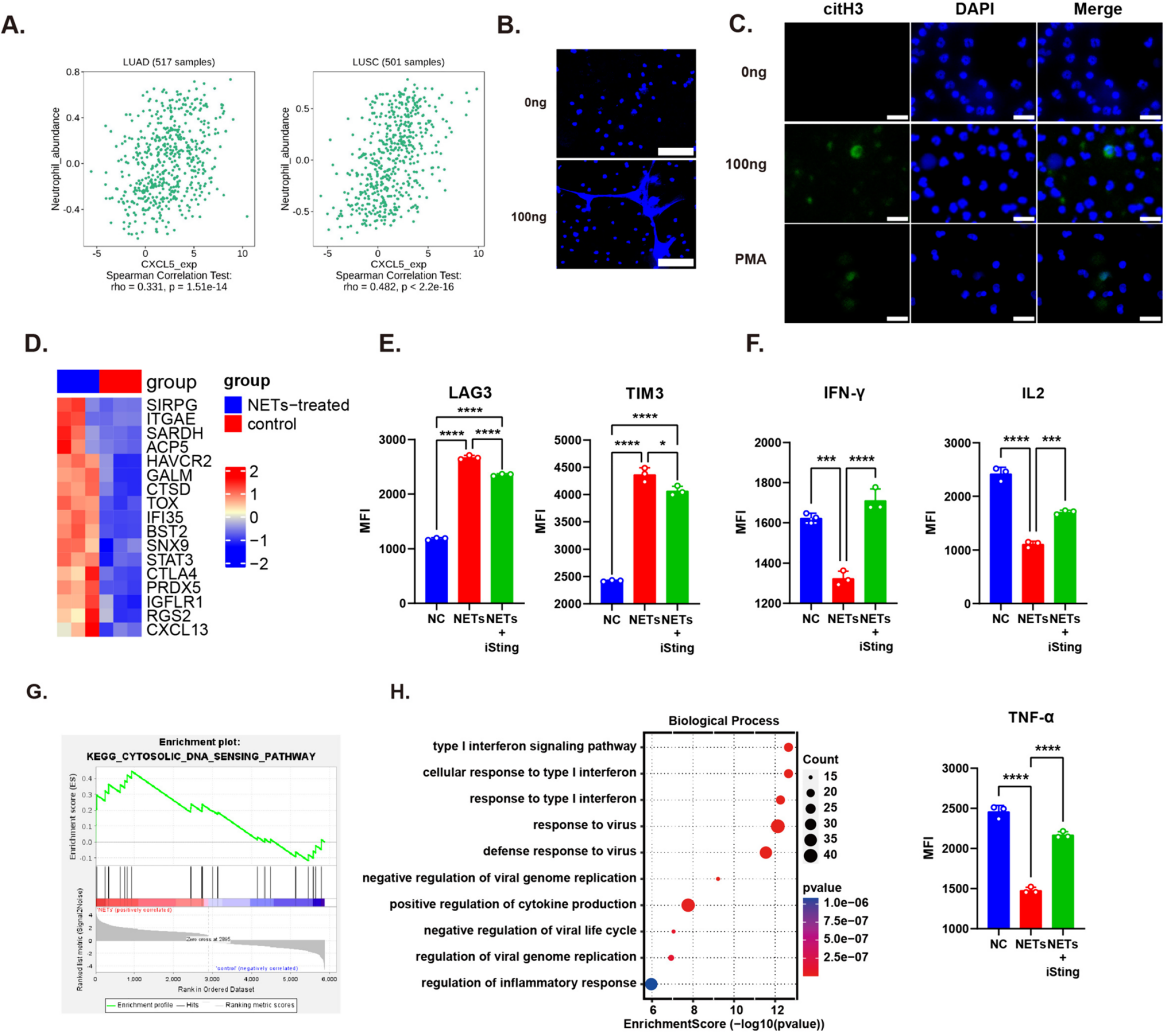
CXCL5 can stimulate neutrophil NETosis which promotes CD8^+^ T cell dysfunction. **a** Correlation between neutrophil abundance and CXCL5 expression. **b** representative view of NETs after stimulation with rhCXCL5 (10 ng/ml), scale bar: 100 μm. **c** Citrullinated histones of neutrophils stimulated by rhCXCL5 (100 ng/ml) and PMA (100 ng/ml), scale bar: 100 μm. **d** Heatmap of exhaustion signature on CD8^+^ T cells without or with NETs treatment. **e** Statistical plot of TIM3 and LAG3 MFI on CD8^+^ T cells. **f** Statistical plot of IFN-γ, TNF-α and IL2 MFI. **g** GSEA enrichment plot of KEGG cytosolic DNA sensing pathway. **h** GO enrichment analysis of RNA-seq of CD8^+^ T cells without or with NETs treatment. All data are mean ± SD and were analyzed by two-tailed, unpaired Student’s *t* test. Data are cumulative results from at least three independent experiments

Studies have demonstrated that CXCL5 can recruit neutrophils that express high levels of the receptor CXCR2. In addition, CXCL5 expression was positively associated with neutrophil abundance in the TCGA LUSC and LUAD datasets (Fig. 5a). Considering that neutrophil NETosis, which releases chromatin outside the cell, is a significant phenomenon reported to be associated with tumor progression [23], we stained neutrophils with the nucleic acid dye SYTOX Green and observed a stained web-like structure under a fluorescent microscope after rhCXCL5 treatment (Fig. 5b). Therefore, we detected citrullinated histone H3, a marker of NETs, in rhCXCL5-treated neutrophils using immunofluorescence and observed a CitH3 fluorescence signal after rhCXCL5 treatment (Fig. 5c). NETs play an important role in tumor metastasis and promote immune evasion, but their influence on immune cells has not been fully elucidated. We further explored their influence on CD8^+^ T cells by co-culturing these cells with freshly isolated NETs. RNA-seq analysis demonstrated that exhaustion-associated genes were upregulated in CD8^+^ T cells after treatment with NETs (Fig. 5d). We observed increased expression of TOX, a transcription factor that regulates immune checkpoint expression, and upregulation of TIM3 and LAG3 (Fig. 5e). However, IFN-γ, TNF-α, and IL2 expression were significantly decreased (Fig. 5f). GSEA and GO enrichment analysis showed a significant upregulation of the cytosolic DNA-sensing pathway and its downstream type I interferon-associated pathway (Fig. 5g, h). STING1 is pivotal in the cGAS-STING cytosolic DNA sensing pathway, and its expression was increased in NETs-treated T cells. Inhibiting STING using its inhibitor C176 significantly upregulated IFN-γ, TNF-α, and IL2 expression but slightly suppressed TIM3 and LAG3 (Fig. 5e, f), suggesting a more complex mechanism that regulates NETs-induced T cell dysfunction. These results indicate that CXCL5 stimulates NETosis, which subsequently promotes CD8^+^ T cell dysfunction, partly through the STING-mediated pathway.

### Blocking A2AR signaling effectively inhibits CXCL5 expression and tumor growth in vivo

**Fig. 6:**
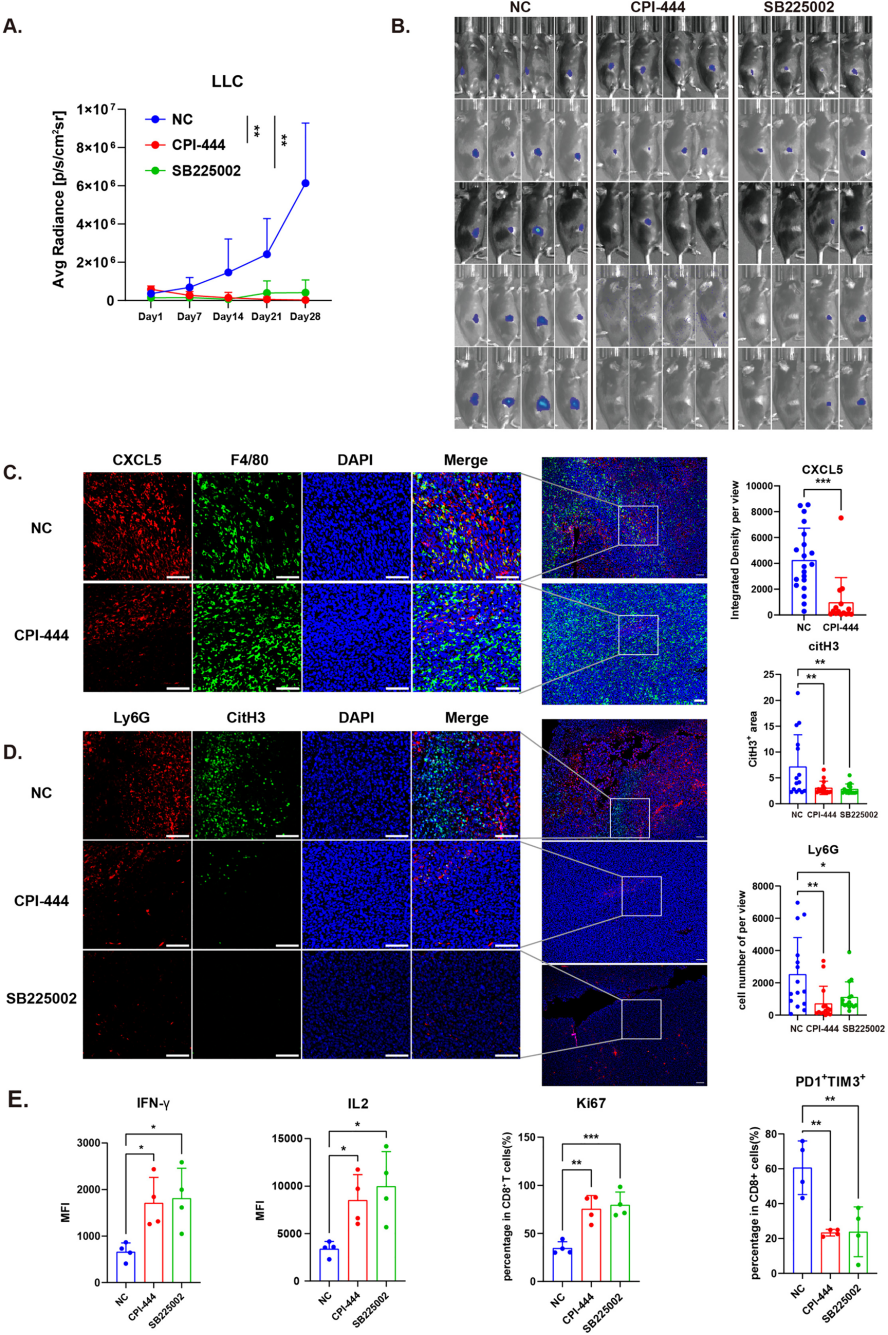
Blocking A2AR signaling can effectively inhibit tumor growth and decrease CD8^+^ TILs dysfunction. **a** The change in average radiance of s.c.-inoculated LLC tumors in C57 mice. **b** Image of LLC-bearing C57 mice under IVIS^®^ Spectrum in Vivo Imaging System. **c** Immunofluorescence image of CXCL5 in LLC tumor tissues (scale bar: 100 μm); each dot represents one view per tumor tissue section, five views per tumor tissue sample, *n* = 5 × 4. **d** Immunofluorescence image of NETs and neutrophils (Ly6G^+^) (scale bar: 100 μm) each dot represents one view per tumor tissue section, five views per tumor tissue sample, *n* = 5 × 4. E. Statistical plot of flow cytometry analysis of tumor-infiltrating CD8^+^ T cells expressing PD1, TIM3, IFN-γ, IL2, and Ki67. All data are mean ± SD, and were analyzed by two-tailed, unpaired Student’s *t* test

To examine the effects of A2AR on CXCL5, neutrophils, and tumor growth in vivo, we constructed a mouse model bearing subcutaneous LLC tumors (1 × 10^6^ cells per mouse). We treated the mice with CPI-444 10 mg/kg (A2AR inhibitor) daily from day 1 after inoculation and SB225002 10 mg/kg (a CXCR2 inhibitor) every three days from day 4 after inoculation until day 28. Tumor growth was monitored weekly. The results demonstrated that the A2AR and CXCR2 inhibitors significantly inhibited tumor growth after treatment (Fig. 6a, b). Some mice were tumor-free after 14 days of treatment. To evaluate the tumor-infiltrating immune cells, we subcutaneously inoculated 2 × 10^6^ LLC tumor cells into 6-week-old mice and treated them with CPI-444 or SB225002 for 20 days. Tumor tissues were analyzed using immunofluorescence and flow cytometry. We found that CXCL5 expression decreased after CPI-444 treatment (Fig. 6c). Neutrophil infiltration and NETs area significantly reduced after CPI-444 and SB225002 administration (Fig. 6d). We tested CD8^+^ T cell function in tumors and found that treatment reduced PD1^+^ TIM3^+^ T cells and rescued IFN-γ and IL2 expression (Fig. 6e). Ki67^+^ T cells increased after treatment (Fig. 6e). These data suggest that A2AR mediates CXCL5 expression in LLC tumors. Blocking A2AR significantly reduced neutrophil infiltration and NETosis.

**Fig. 7:**
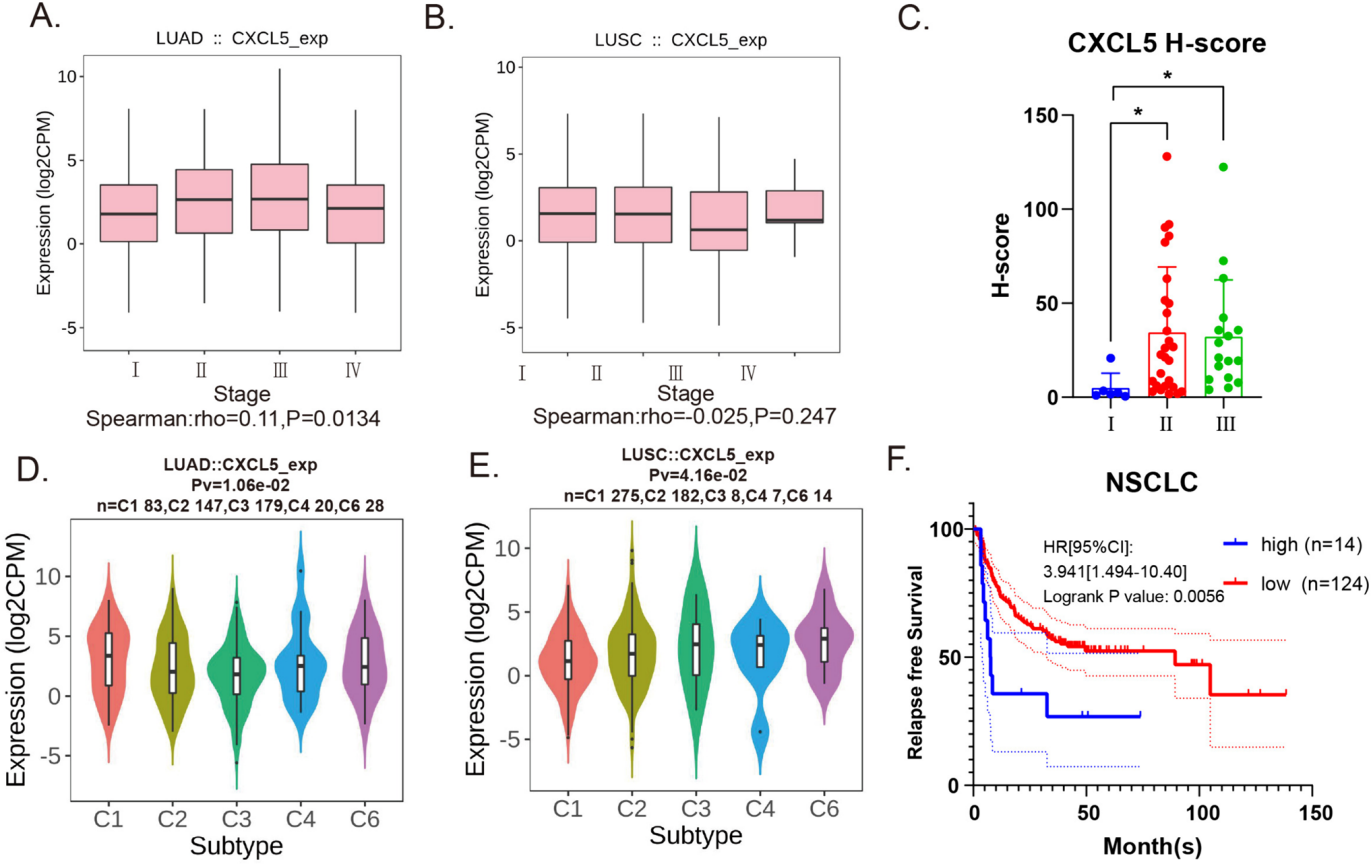
CXCL5 expression in TCGA database and its correlation with clinical data.** a**, **b** CXCL5 expression in TCGA tumor tissues at different clinical stages. **c** Statistical plot of H-scores of TMA CXCL5 expression. **d**, **e** CXCL5 expression in TCGA NSCLC tissues classified by immune infiltration status. **f** Kaplan–Meier plot of CXCL5 of patients with NSCLC

### CXCL5 is associated with poor prognosis in patients with NSCLC

To evaluate the relationship between CXCL5 and clinical data, we used TISIDB (http://cis.hku.hk/TISIDB/index.php) to analyze CXCL5 expression in NSCLC tissues classified by clinical stage in the TCGA database and found that CXCL5 expression was positively associated with clinical stage in LUAD but not LUSC (Fig. 7a, b). Additionally, we analyzed NSCLC tissue microarrays and observed higher CXCL5 expression in tissues with pathologically grades II and III (Fig. 7c). Thorsson et al. characterized tumor immune microenvironment and identified six immune subtypes: Wound Healing (C1), IFN-γ Dominant (C2), Inflammatory (C3), Lymphocyte Depleted (C4), Immunologically Quiet (C5), and TGF-β Dominant (C6) [24]. The C6 was the least favorable of the six subtypes in terms of prognosis and expressed higher levels of CXCL5 (Fig. 7d, e). Furthermore, the C6 immune subtype of NSCLC which has been reported to have a negative correlation with immunotherapy expressed higher levels of CXCL5 (Fig. 7d, e). Kaplan–Meier analysis generated from PrognoScan (http://dna00.bio.kyutech.ac.jp/PrognoScan/) demonstrated that high expression of CXCL5 was associated with decreased survival of NSCLC patients (Fig. 7f). This suggests that NSCLC with high CXCL5 expression is associated with a poor prognosis.

## Discussion

Previous studies regarded the TME as a significant determinant of immunotherapy efficacy [25]. Many efforts have been made to characterize the cellular components of the TME, and TAMs have gained much attention because they account for a large proportion of tumor-infiltrating immune cells in the TME. By simulating the Th1 and Th2 immune responses, TAMs can be divided into M1 pro-inflammatory and M2 anti-inflammatory types [26]. However, recent studies have illustrated the inadequacy of this dichotomy in depicting tumor-associated macrophage biology. TAMs are well-characterized sources of cytokines in TME. TAMs-derived cytokines directly promoted cancer metastasis [11, 27, 28]. In our study, NSCLC cells induced CXCL5 upregulation in TAMs which recruited neutrophils and induced NETosis. NETs promoted CD8^+^ T cell dysfunction and an exhausted-like phenotype, significantly inhibiting antitumor immunity.

Metabolite-mediated interactions between tumor cells and TAMs are pivotal for the formation of the immunosuppressive TME [29]. The conversion of ATP to adenosine has been demonstrated to be very active in the TME, while the expression of the ectonucleotidases CD39 and CD73 is high on the tumor and stromal cell surfaces [30]. The adenosine receptor A2AR has a high affinity for extracellular adenosine and subsequently suppresses immune effector cells while activating regulatory cells [31–33]. Additionally, it can stimulate the activation of A2AR in macrophages to promote the expression of immunosuppressive cytokines, consequently promoting the formation of an immunosuppressive microenvironment [18]. We discovered that the ectonucleotidases CD39 and CD73 were expressed on macrophages and tumor cells, respectively. This is in line with a previous study on hepatocellular carcinoma that observed the macrophage CD39 and HCC cell CD73 synergistically activate ATP–adenosine pathway to directly impair antitumor immunity [34]. In our study, we found that this synergistic effect upregulated the CXCL5 expression in macrophages by activating A2AR, subsequently inducing NETosis and promoting CD8^+^ T cell dysfunction. Additionally, CD73^+^ macrophages have been documented in particular tissues like the peritoneum and glioma [35, 36]. These findings suggest the importance of investigating the involvement of CD73 across diverse tumor types and immune cell populations.

CXCL5 is generated from tumor cells in some types of cancers [37], stromal cells including macrophages [38], cancer-associated fibroblasts [39], and mesenchymal stem cells [40]. High CXCL5 expression in hepatocellular carcinoma promotes tumor progression and mediates neutrophil infiltration [41]. In gastric cancer, macrophage-derived CXCL5 promotes tumor cell migration through the CXCR2/STAT3 pathway [27] and facilitates chemoresistance via the CXCL5/PI3K/AKT/mTOR pathway [42]. CXCL5 mediated apoptosis and autophagy in AURKA-overexpressing NSCLC [43] cells. CXCL5-induced neutrophil accumulation inhibits CD8^+^ T cell function [44]. In our study, we found that CXCL5 stimulated NETosis, which promoted CD8^+^ T cell dysfunction. Additionally, there is difference in the rho value of the correlation between CXCL5 and immune cell infiltration in LUAD and LUSC (Supplementary Fig. 1e and main Fig. 5a). The differences in correlation strength may be attributed to the heterogeneous immune landscape and the distinct intrinsic signaling [45, 46], suggesting different cell-to-cell interaction modes within TME of LUAD and LUSC.

A2AR signaling is reported to suppress NFκB activation in T lymphocytes by stimulating CREB [47]. However, we found that blockade of A2AR inhibited phosphorylation of NFκB subunit P65, and A2AR agonist upregulated phosphorylated NFκB expression. In line with our study, researchers have demonstrated that the A2AR antagonist caffeine significantly suppressed P65 phosphorylation in macrophages [48], while the agonist CGS21680 promoted macrophage M2 polarization and increased P65 expression [49]. These results suggest that A2AR may demonstrate different regulation modes in modulating NFκB activation.

Previous studies have shown that neutrophils extrude NETs in the context of the tumor microenvironment and exert pro-tumor effects through various mechanisms, including angiogenesis, ECM degradation, and metabolic switching. Studies have shown that many inflammatory factors can induce NETs release such as IL8 [50] and HMGB1 [51]. NETs contain multiple components that influence immune cells and indirectly promote tumor progression. For example, NETs promote CD8^+^ T cell exhaustion via embedded PDL1 [52]. In our study, we observed that NETs upregulate exhaustion-related genes on CD8 ^+^ T cells, potentially via the STING pathway. Inhibition of STING slightly downregulated TIM3 and LAG3 expression but significantly upregulated cytokine expression, indicating a more complicated mechanism involved in NETs-induced CD8^+^ T cell exhaustion. However, further studies are required to confirm this regulatory mechanism.

This study demonstrated that A2AR signaling mediated interaction between lung cancer cells and macrophages through NFκB, which regulated macrophage-derived CXCL5 expression. The differential expression of CD39 and CD73 further suggested the existence of tumor cell-macrophage interactions. CXCL5 induced NETosis in neutrophils and promotes CD8^+^ T cell dysfunction.

### Supplementary Information

Below is the link to the electronic supplementary material.

Supplementary file 1 (DOCX 3385 kb)

Supplementary file 2 (XLSX 1107 kb)

Supplementary file 3 (XLSX 15 kb)

Supplementary file 4 (XLSX 10 kb)

Supplementary file 5 (XLSX 10 kb)

Supplementary file 6 (XLSX 11 kb)

Supplementary file 7 (XLSX 56 kb)

## Data Availability

Data sharing in this study is available from the corresponding author upon reasonable request.

## Abbreviations

A2AR: Adenosine receptor 2a
IFN-γ: Interferon gamma
IL-2: Interleukin-2
NETs: Neutrophil extracellular traps
NSCLC: NON-small cell lung cancer
PD-1: Programmed cell death 1
qRT-PCR: Quantitative real-time PCR
shRNA: Short hairpin RNA
TAMs: Tumor-associated macrophages
TMA: Tissue microarrays
TME: Tumor microenvironment
TNF-α: Tumor necrosis factor alpha
