# Supplementary material for: A2AR-mediated CXCL5 upregulation on macrophages promotes NSCLC progression via NETosis
Source: Cancer Immunol Immunother. 2024 Apr 20;73(6):108. doi: 10.1007/s00262-024-03689-3 (PMC11032303; doi:10.1007/s00262-024-03689-3)

### ****Supplementary figure 1. Co-culture of macrophages and NSCLC cells****

A. Macrophages and H460 were co-cultured using a transwell device (0.4μm). B. Purity of CD14^+^ cells before and after magnetic sorting. C. Heatmap of differentially expressed genes comparing untreated and co-cultured macrophages. D. GO enrichment of DEGs in macrophages. E. Correlation of CXCL5 and macrophages in NSCLC. F-G. Gating strategy of macrophages and NSCLC cell lines in imaging flow cytometry.

### ****Supplementary figure 2. RNA-seq analysis of H460****

A. Heatmap of DEGs comparing treated and untreated H460. B. KEGG enrichment of DEGs on H460. C. GO enrichment of DEGs on H460.

### ****Supplementary figure 3. Correlation of CXCL5 with Top 10 DEGs****

A. Correlation of CXCL5 with membrane expression genes in the top 10 DEGs list in LUSC. B. Correlation of CXCL5 with membrane expression genes in the top 10 DEGs list in LUAD.

### ****Supplementary figure 4. GSEA analysis of macrophages and gating strategy****

A. GSEA analysis of GO CAMP metabolic processes on macrophage DEGs. B. A2AR expression on untreated and co-cultured macrophages. C. Gating strategy of flow cytometry for detecting A2AR on macrophages. D. CD163 expression on untreated and co-cultured macrophages. E. Expression of CXCL5 after macrophages were treated with A2AR inhibitor. F. Gating strategy of imaging flow cytometry for detecting A2AR and CD163 on macrophages.

### Supplementary figure 5. Gating strategy of imaging flow cytometry detecting NFκB translocation.

### ****Supplementary figure 6. LLC tumors and gating strategy****

1. Tumor volume of subcutaneously inoculated LLC. B-C. Gating strategy of CD8^+^ TILs.

### ****Supplementary figure 7. Kaplan-Meier analysis base on median and quartile cutoffs****


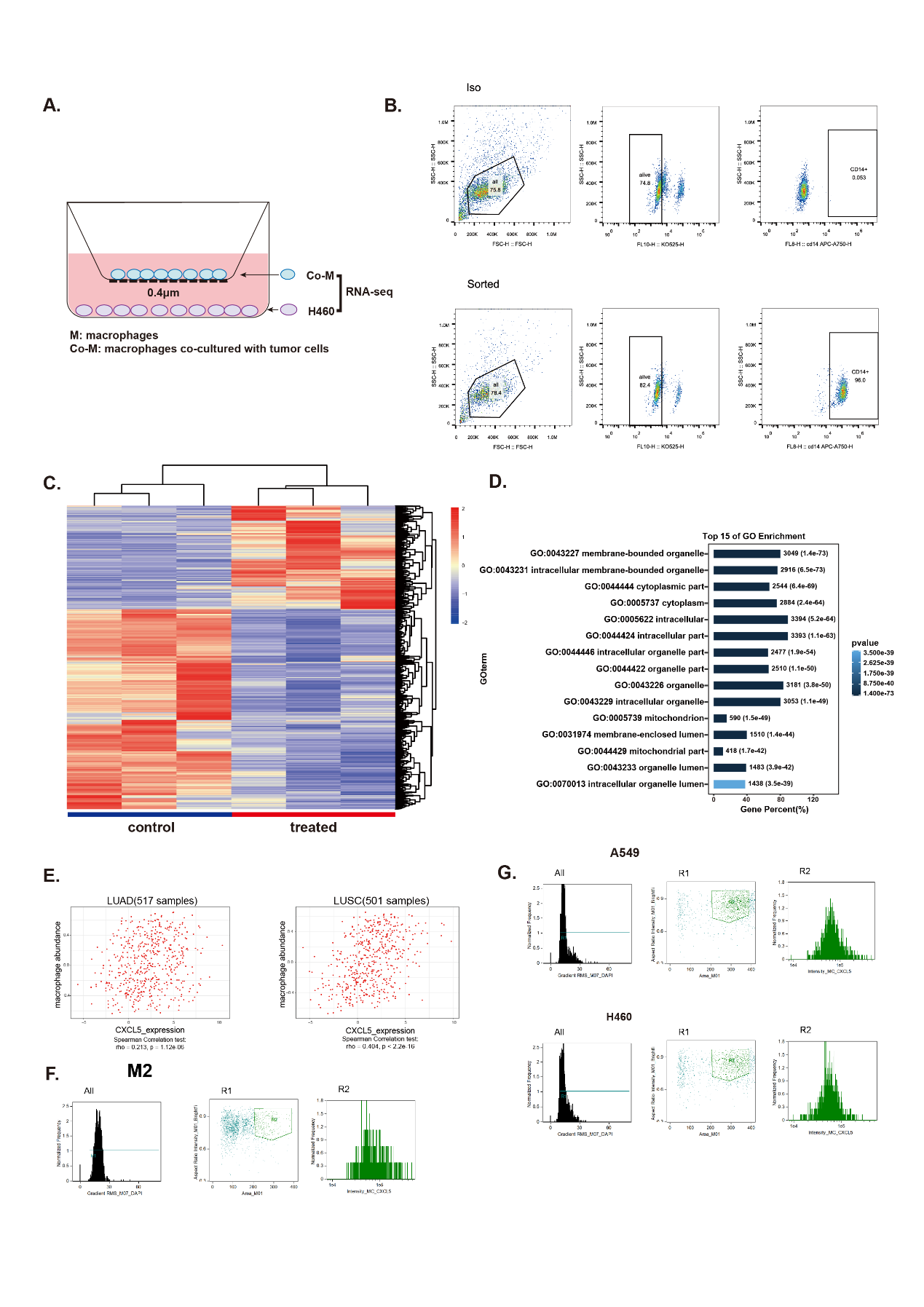

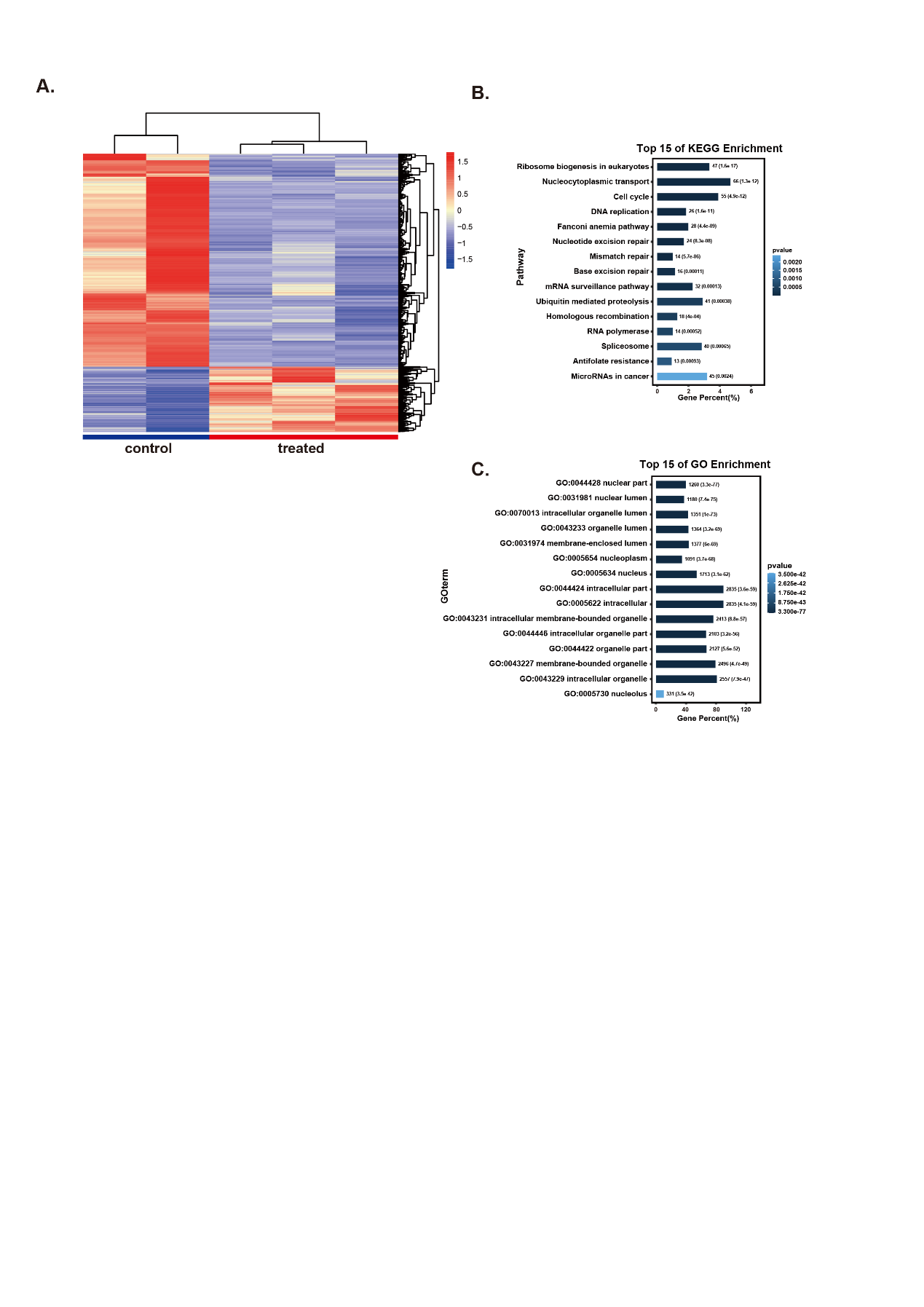

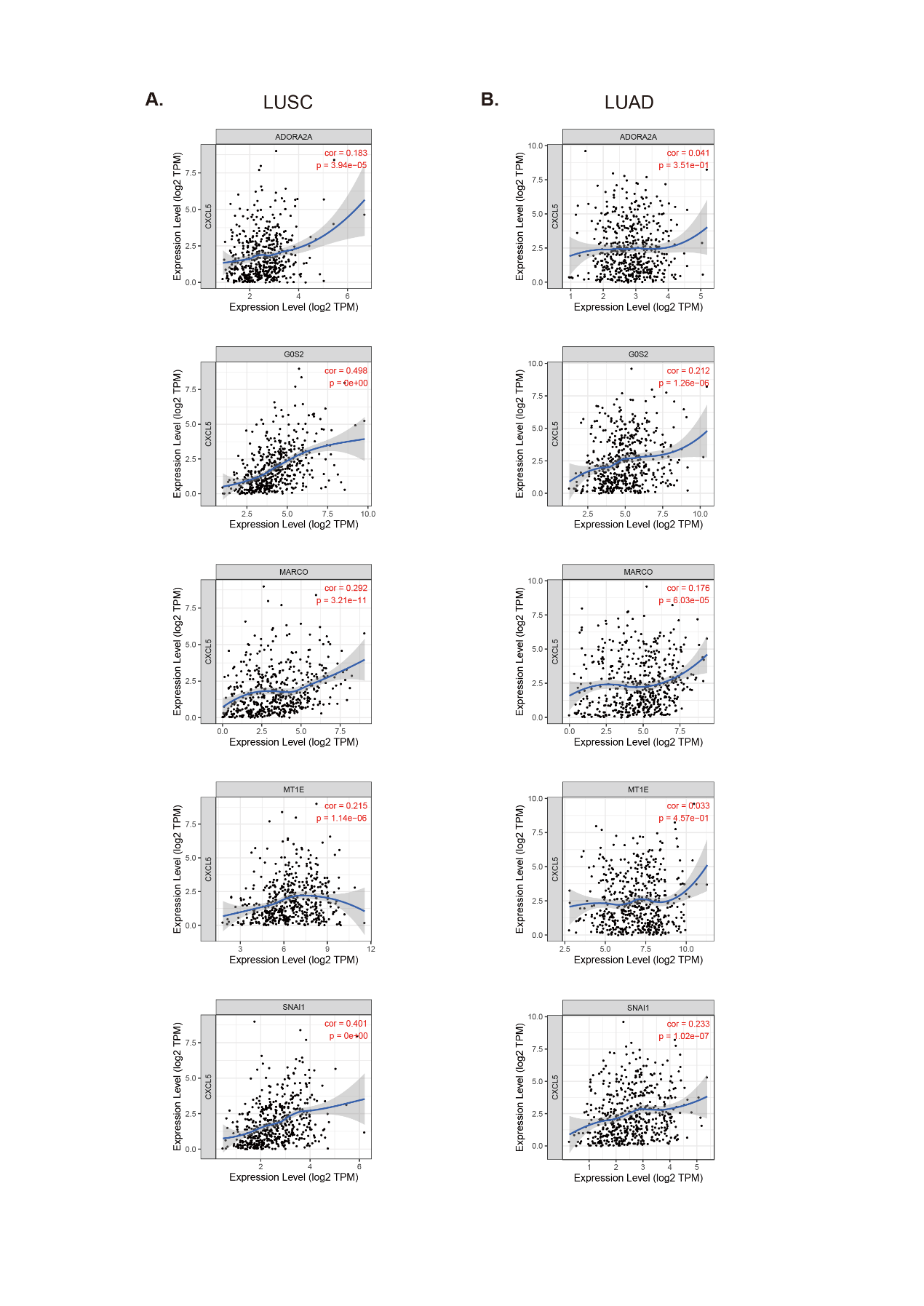

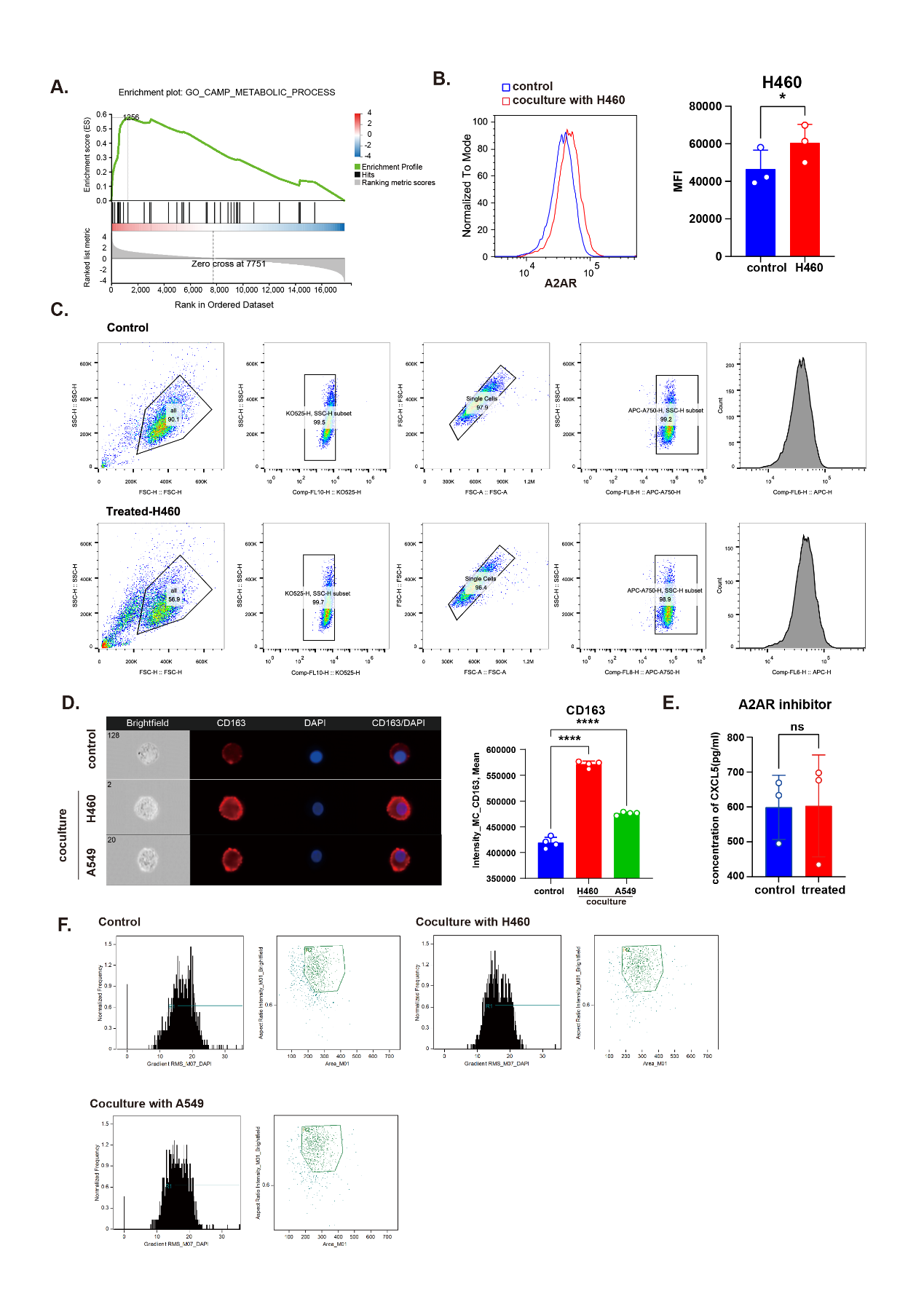

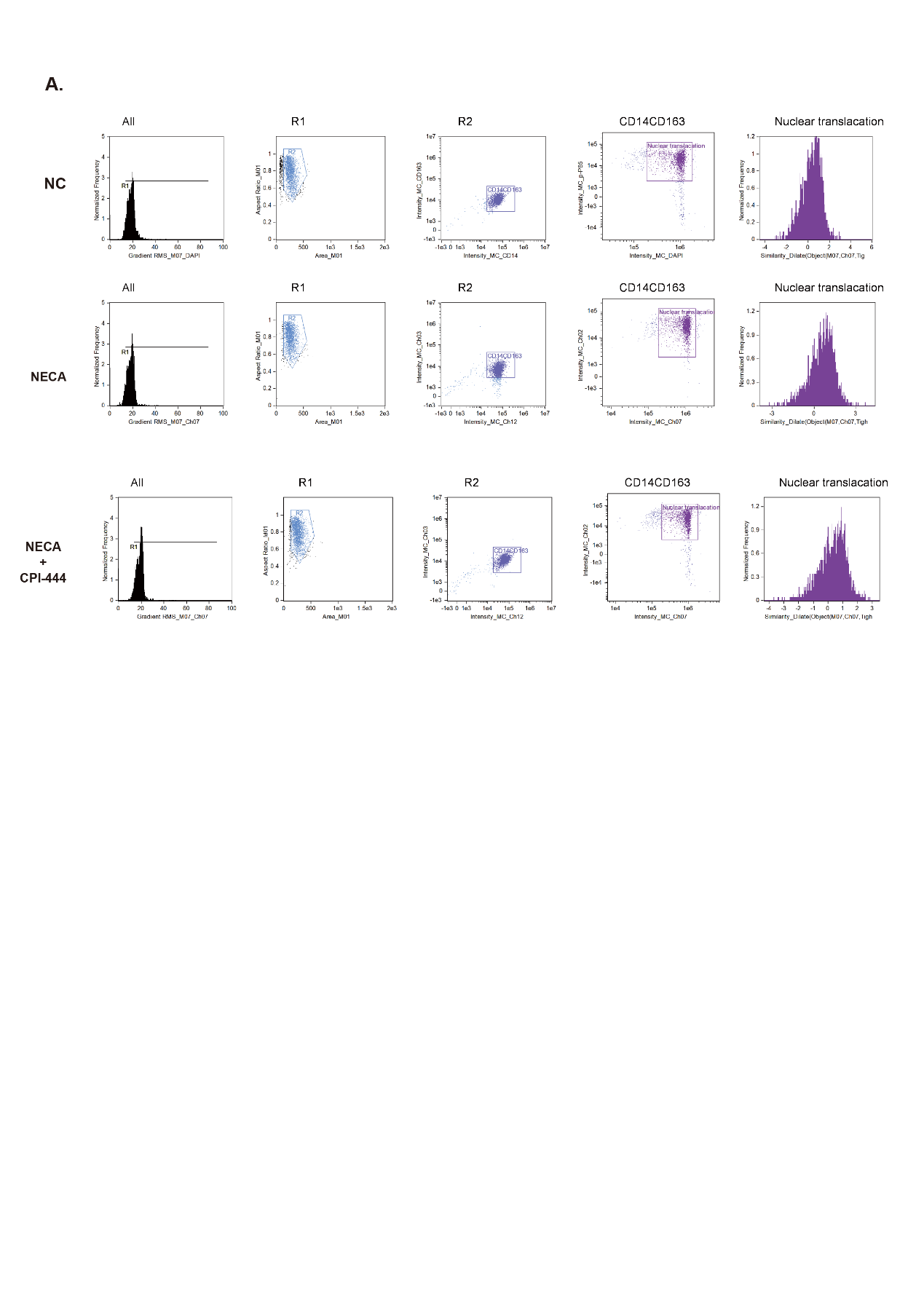

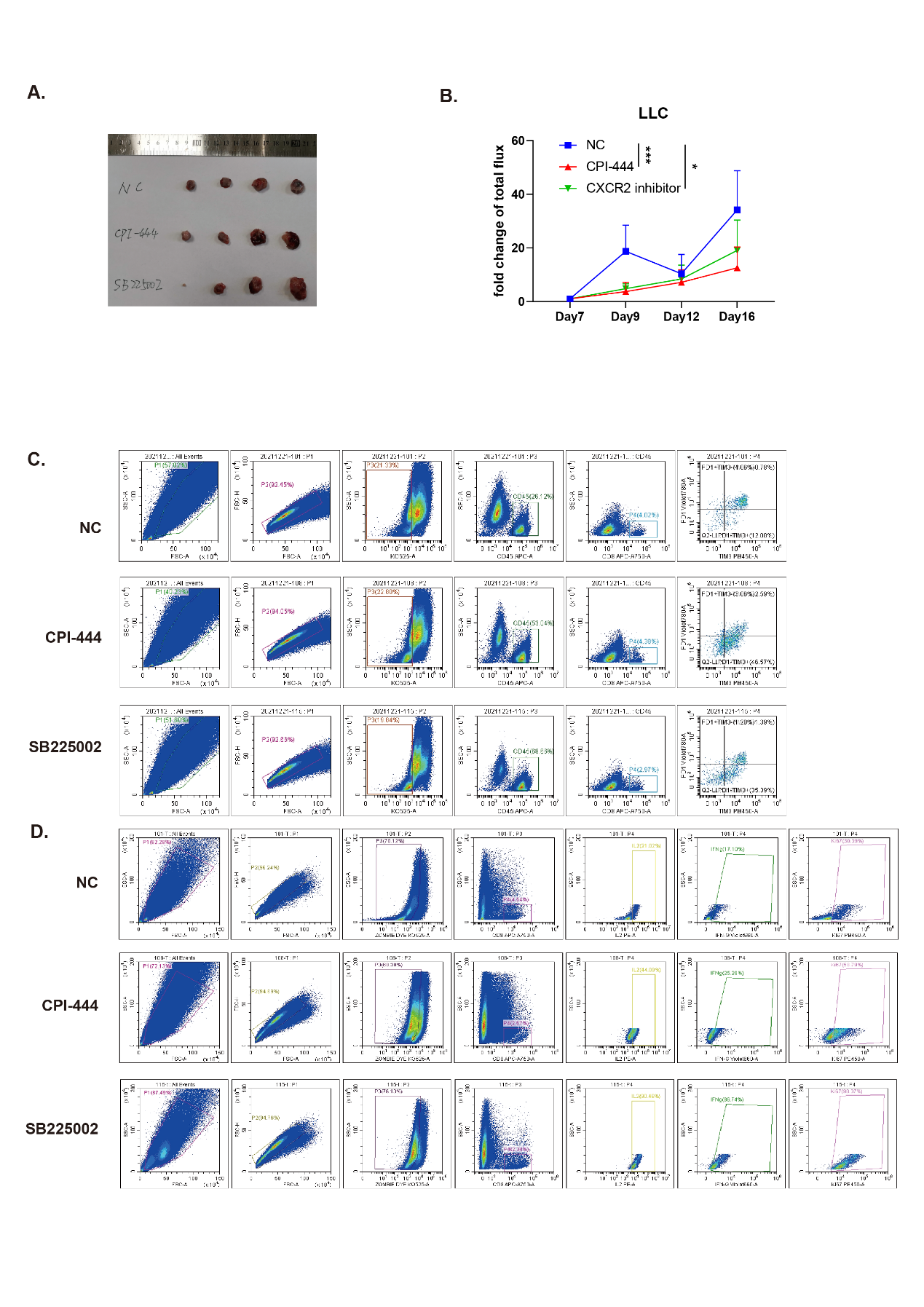


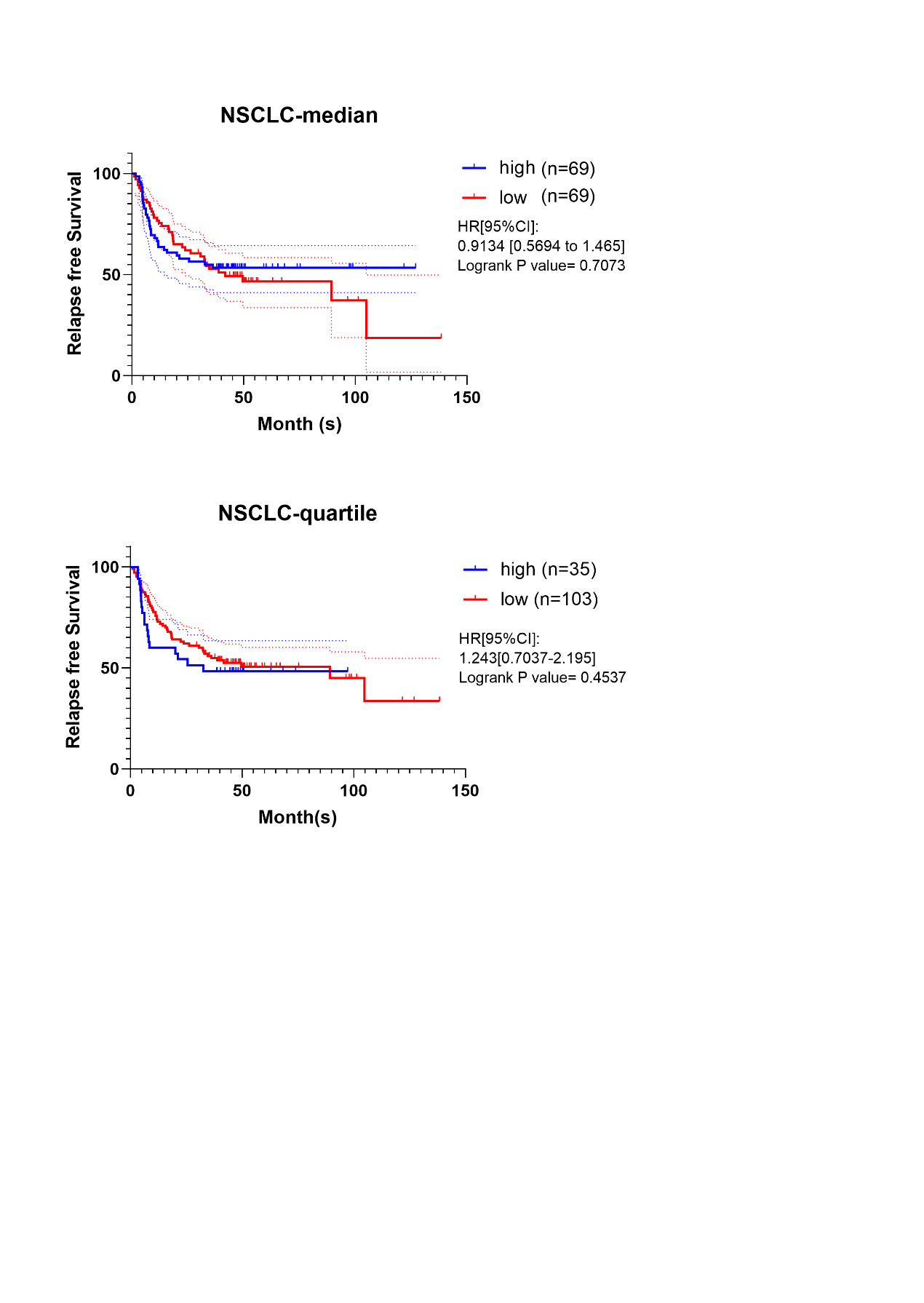

Supplement: Supplementary file 1 — (DOCX 3385 kb) [file 262_2024_3689_MOESM1_ESM.docx]
